# A process for producing lignin and volatile compounds from hydrolysis liquor

**DOI:** 10.1186/s13068-017-0729-9

**Published:** 2017-02-23

**Authors:** Tooran Khazraie, Yiqian Zhang, Dmitry Tarasov, Weijue Gao, Jacquelyn Price, Nikolai DeMartini, Leena Hupa, Pedram Fatehi

**Affiliations:** 1 Johan Gadolin Process Chemistry Center, Laboratory of Inorganic Chemistry, Åbo Akademi University, Piispankatu 8, 20500 Turku, Finland; 2 Chemical Engineering Department, Lakehead University, 955 Oliver Road, Thunder Bay, ON P7B 5E1 Canada; 3 Bioeconomy Technology Centre, FPInnovations, 2001 Neebing Ave, Thunder Bay, ON P7E 6S3 Canada

**Keywords:** Acidification, Hydrolysis, Lignin, Furfural, Biorefining

## Abstract

**Background:**

Hot water hydrolysis process is commercially applied for treating wood chips prior to pulping or wood pellet production, while it produces hydrolysis liquor as a by-product. Since the hydrolysis liquor is dilute, the production of value-added materials from it would be challenging.

**Results:**

In this study, acidification was proposed as a viable method to extract (1) furfural and acetic acid from hot water hydrolysis liquor and (2) lignin compounds from the liquor. The thermal properties of the precipitates made from the acidification of hydrolysis liquor confirmed the volatile characteristics of precipitates. Membrane dialysis was effective in removing inorganic salts associated with lignin compounds. The purified lignin compounds had a glass transition temperature (Tg) of 180–190 °C, and were thermally stable.

**Conclusions:**

The results confirmed that lignin compounds present in hot water hydrolysis liquor had different characteristics. The acidification of hydrolysis liquor primarily removed the volatile compounds from hydrolysis liquor. Based on these results, a process for producing purified lignin and precipitates of volatile compounds was proposed.

**Electronic supplementary material:**

The online version of this article (doi:10.1186/s13068-017-0729-9) contains supplementary material, which is available to authorized users.

## Background

The current low price of pulp products has significantly hampered the overall profitability of the pulping industry. Forest biorefinery has been considered as an option to revisit this industry. In forest biorefinery, energy or biomaterials are produced in addition to pulp products [1]. In a forest biorefining process, cellulose can be produced as dissolving pulp, while lignin and hemicelluloses are converted to other value-added products [1, 2].

Hot water hydrolysis process is commercially used for extracting hemicelluloses from wood chips. It was reported that hydrolysis liquor contains hemicelluloses and some lignin that can be used in the production of value-added products [3]. Hot water hydrolysis process can be carried out prior to pulping process [4, 5] or in wet torrefaction process [6]. In both processes, the hydrolyzed wood chips are transferred to the next steps for producing dissolving pulp or wood pellets, while a large volume of hydrolysis liquor that contains dissolved hemicelluloses and lignin is treated as a waste [7].

It is well-known that the severity of hydrolysis conditions affects the removal of lignocelluloses from wood chips, as well as the properties of extracted hemicellulose and lignin [5], which implies that changing the hydrolysis process conditions allows for producing hydrolysis liquor with different properties and chemistries. The first objective of this work was to study the effect of hydrolysis conditions in extracting lignocelluloses from the wood chips.

The hydrolysis liquor can be used as fermentation intermediate for the production of xylitol and ethanol due to the presence of dissolved hemicelluloses in the hydrolysis liquor [8]. However, fermentation inhibitors, such as furfural, acetic acid, and lignin derivatives in the hydrolysis liquor would hamper the efficiency of the fermentation processes [9, 10]. Lignin can also be used as fuel or in the production of phenols, for instance [11]. Presently, hemicellulose and lignin of the hydrolysis liquor cannot be economically utilized in the production of value-added products due to the dilute nature of the hydrolysis liquor. In the past, ultrafiltration was proposed for isolating hemicellulose and lignin from hydrolysis liquor [12, 13], which could be efficient in concentrating hydrolysis liquor. However, filter blockage and fouling should be considered as main operation challenges of this process [14–16]. Adsorption and flocculation can also be viable options for extracting hemicellulose and lignin from hydrolysis liquor, but the need for recovering adsorbents, the high price and/or sensitivity of flocculants to the chemistry of hydrolysis liquor may be major barriers in the implementation of adsorption or flocculation process at an industrial scale [17]. Acidification has been commercialized as LignoBoost and LignoForce technologies for extracting kraft lignin from black liquor of kraft pulping process [18, 19]. Although some research results showed that acidification was efficient in extracting organics from hydrolysis liquor of kraft-based dissolving pulp production process [20–22], it is not clear what components were extracted via acidifying hydrolysis liquor and what properties the extracted components had. Since the literature results on the acidification process are not conclusive, the second objective of this work was to evaluate the effectiveness of acidification in extracting different components from a hydrolysis liquor.

In the present work, spruce wood chips were treated with hot water in a pulping digester and then the hydrolysis liquor was collected and characterized to specify the impact of acidification on the precipitation of organic compounds extracted from wood chips.

Subsequently, the thermal properties of the precipitates were investigated in order to identify the potential end-use applications for the precipitates. The main novelties of this work were as follows:(1) a detailed investigation on the efficiency of acidification in removing different organic components from hydrolysis liquor including volatile compounds and hydrolysis lignin, which would impact the properties of the precipitates and their end-use applications, and (2) the development of a process for producing hydrolysis lignin and precipitates made of volatile compounds.

## Methods

### Materials

Industrially produced spruce wood chips (with the moisture content of 37%) were received from a mill located in northern Ontario, Canada. This wood species is commonly used in Finland and Canada for pulp and energy production purposes. Sodium hydroxide pellets, sodium sulfate (analytical grade), sodium sulfite (analytical grade), acetic anhydride, para-hydroxybenzoic acid, 2-chloro-4,4,5,5-tetramethyl-1,2,3-dioxaphospholane, 3-(trimethylsilyl) propionic-2,2,3,3-d_4_ acid sodium salt (TSP), chromium(III) acetylacetonate, deuteriochloroform (CDCl_3_), anhydrous pyridine, hydrochloric acid (37%, reagent grade), and sulfuric acid (98 wt%) were received from Sigma Aldrich company. Cellulose acetate membrane dialysis with a molecular cut-off of 1000 g/mol was obtained from Wako Chemicals, Japan.

### Hot water hydrolysis treatment

In this set of experiments, 300 g of wood chips was placed in a 2 L pulping digester, Greenwood, TX. The impact of liquid to solid (L/S) ratio in the autohydrolysis of softwood was studied in the past [23, 24]. Leppanen et al. conducted the autohydrolysis of Norway spruce with L/S ratio 15/1 [25]. In current work, due to the small size of the pulping digester (2 L), the L/S ratio of 15/1 generated hydrolysis liquor with undetectable organic compounds (as it would result in a small quantity of wood in the digester). Therefore, a liquid to wood ratio of 8 (on a dried basis) was selected in this analysis with adding deionized water to the digester. The heating rate of the hydrolysis treatment was adjusted to 4.5 °C/min when the temperature of the digester was below 100 °C and to 2.5 °C/min when the temperature of the digester increased above 100 °C. The liquor in the digester was circulated at the flow rate of 6 L/min. Furthermore, autohydrolysis was successfully applied to spruce wood chips in the temperature range of 100–240 °C and time of 100 min [24, 25]. Song et al. reported a significant lignin isolation in the temperature range of 160–180 °C [24, 26]. In the current work, the hydrolysis treatment was conducted at 170, 180, or 190 °C for 15 or 45 min, where the pH of hydrolysis liquor after the hydrolysis treatment (i.e., after experiment at room temperature) was 3.2–3.6.

**Fig. 1:**
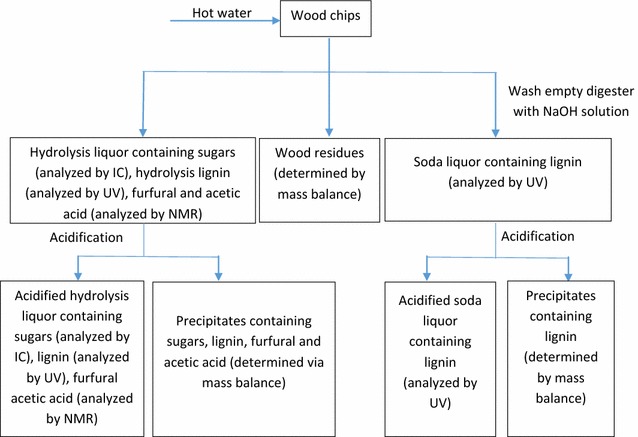
Experimental procedure conducted for producing hydrolysis liquor and analytical methods followed for analyzing the products

Figure 1 shows the flow chart of the experimental procedure conducted in this work to produce hydrolysis liquor and the methods to analyze it. As seen, after hot water hydrolysis treatment, the hydrolysis liquor was collected from the digester and digester was made empty. The lignin of hydrolysis liquor was denoted as hydrolysis lignin. Acid-soluble lignin with different structures is extracted during hot water hydrolysis process [12, 15]. In hydrolysis liquor, some lignin components are very hydrophilic and are soluble under strong acidic conditions (e.g., pH of 2), but some others have more complex structures, and are probably more hydrophobic and thus less soluble under acidic conditions. Furthermore, Lawoko et al. [27] and Lappane [25] stated that lignin compounds in spruce formed lignin–carbohydrate complexes (LCC) [27]. Therefore, it is possible that lignin with different structures and LCC compounds were formed in the hydrolysis process. In the present study, it was noted that a part of lignin compounds adsorbed on the interior parts of the digester. To collect adsorbed lignin from the interior surface of digester, the digester (after collection of treated wood chips and hydrolysis liquor) was treated with 1.5 L of NaOH solution (3 wt%), which is donated as a soda liquor in this work. This soda treatment was repeated at different temperatures of 80 and 90 °C for 0.5, 1, and 1.5 h to collect the adsorbed lignin from the digester. The lignin dissolved in the soda liquor from treating the digester is denoted as soda liquor lignin in this work. The concentration of soda liquor lignin in the soda liquor was analyzed as stated in the following section. The treatment at 90 °C for 1 h generated a soda liquor with the highest lignin concentration. To ensure that no lignin had been remained in digester after soda liquor treatment, the treated digester was treated with fresh soda liquor solution as stated above, and the analysis confirmed negligible lignin in this solution. Therefore, the soda liquor treatment at 90 °C for 1 h with 3 wt% NaOH solution was selected as a method for extracting soda liquor lignin from the digester.

### Chemical composition analysis

In this set of experiments, spruce wood chips were dried and then ground to a size smaller than 1 mm and then kept in a desiccator prior to analysis. In order to measure the hemicellulose and cellulose contents, the NREL method was applied to the ground wood particles [28]. The Klason lignin method was used to determine the contents of acid-soluble and acid-insoluble lignin of the ground wood particles (T 222 om-98 and UM 250). To determine the total content of extractives in spruce wood chips, they were treated with acetone/water (95/5 v/v) in Glas-Col Combo Mantle extraction apparatus for 6 h. The final content of the extractives was determined using a gravimetric method.

### Sugar analysis

The concentrations of polysugars and monosugars in the hydrolysis liquor and soda liquor were determined using ion chromatography, Dionex, ICS 5000, Thermofisher Scientific, equipped with CarboPac™ SA10 column and an electrochemical detector (ED) (Dionex-300, Dionex Corporation, Canada). Deionized water and KOH Eluent Generator (EGC 500 KOH, ThermoScientific) were used to generate an eluent of 1.00 mM of KOH at a flow rate of 1.2 mL/min. The column temperature was set at 30 °C. The monosugar concentration in the liquors was measured without pretreating the liquors but after adjusting pH of the liquors to 7. The hydrolysis and soda liquors were acid-hydrolyzed under the conditions of 4% sulfuric acid at 121 °C for 1 h in an oil bath (Hakke S45, Instruments, Inc., Portsmouth, N.H., USA) based on the method described in the literature [12]. This acid hydrolysis is widely used for converting oligosugars to monosugars [4, 12]. Afterward, the concentration of monosugars in the hydrolysis liquors was measured as stated above, and it reflected the concentration of total monosugars (after conversion of oligosugars to monosugars) in the hydrolysis and soda liquors. The concentrations of polysugars were determined via subtracting total sugar concentrations from monosugar concentrations.

### Lignin, furfural, and acetic acid analyses

The lignin content of the liquors was determined according to TAPPI UM 250 using UV spectrophotometry at 205 nm (GENESYS 10S UV–Vis, Thermo Scientific) [12].

To measure the contents of furfural and acetic acid, the liquors were first dried. Then 0.4 wt% of 3-(trimethylsilyl) propionic-2,2,3,3-d_4_ acid sodium salt (TSP) in deuterium oxide (D_2_O) was prepared. About 40 mg of dried liquor was added to 700 µL of the D_2_O solution. After mixing the solution, it was transferred to the NMR vials. A proton nuclear magnetic resonance, NMR, Varian Unity Inova 500 MHz spectrometer was used for determining the concentrations of furfural and acetic acid in the hydrolysis and soda liquors according to the previously established method [4].

### Molecular weight analysis of hydrolysis and soda liquors

Samples with a 5 g/L concentration in 0.1 mol/L NaNO_3_ were prepared from the hydrolysis and soda liquors, and then they were stirred at 300 rpm for 24 h. Then samples were filtered with a 0.2 µm nylon filter (13 mm diameter), and the filtered solutions were used for molecular weight analysis. The molecular weight of the samples was measured using a gel permeation chromatography, Malvern GPCmax VE2001 Module + Viscotek TDA305 with multi-detectors. The columns of PolyAnalytic PAA206 and PAA203 were used in the analysis, and a 0.1 mmol NaNO_3_ solution was used as solvent and eluent. The flow rate was set at 0.70 mL/min, while the column temperature was 35 °C and poly (ethylene oxide) was used as a standard sample. The UV detector at 280 nm wavelength was used for determining the molecular weight of lignin, and IR detector was used for measuring the molecular weight of polysugars. This method was used for determining the molecular weight of lignin in the past [29].

### Acidification of hydrolysis and soda liquors

Strong sulfuric acid treatment of hydrolysis liquor was followed as a method to separate hydrolysis lignin from hydrolysis liquor. The acidification at a high temperature is commercially used in the LignoForce technology to extract kraft lignin from black liquor [14]. To understand the efficiency of acidification process in isolating lignin from hydrolysis and soda liquors, the liquors were acidified with sulfuric acid (1 mL of 98 wt% in 900 mL liquor) to the pH of 1.5 (Fig. 1). The dilution factor caused by mixing acid with the liquors was considered in determining the concentration of organic compounds in the liquors. Then the mixtures were heated to 80 °C and kept for 15 min at 80 °C. The precipitates formed in hydrolysis and soda liquors were then separated via filtration/centrifugation (4000 rpm for 10 min). The collected samples after centrifugation were analyzed comprehensively. A part of the collected precipitates of soda liquor was dialyzed for 2 days using membrane dialysis, while changing water every 4 h to remove impurity, and the properties of dialyzed samples were assessed.

### TGA and DSC analyses of precipitates

The thermal characteristics of the precipitates made from hydrolysis and soda liquors were analyzed using a thermogravimetric analyzer (TGA) and differential scanning calorimeter (DSC). In this set of experiments, 8–12 mg of dried precipitates were loaded in a platinum (Pt) crucible of a thermogravimetric analyzer (TGA)-i1000 series (Instrument Specialist Inc.) and heated isothermally at 100 °C for 10 min to ensure moisture removals. Then the samples were heated to 700 °C under nitrogen (35 mL/min) with an increment rate of 10 °C/min.

Moreover, the thermal behavior of precipitates were investigated using a differential scanning calorimeter (DSC), TA instrument, Q2000, and the standard cell RC mode of DSC was also used for analysis. The samples were treated at 60 °C in an oven for removing moisture, then 8–10 mg of the dried samples were loaded into a Tzero aluminum pan, and analyzed by heat/cool/heat method in a temperature range from 30 to 250 °C at 50 mL/min in nitrogen. The heating and cooling rates were both controlled at 5 °C/min, and the second heating cycle (showed as exotherm up) was chosen for glass transition and melting point analyses.

## ^31^P NMR analysis of precipitates

The OH functional groups of the precipitates were analyzed by quantitative phosphorous nuclear magnetic resonance (^31^P NMR) analysis. This process was carried out following a previously established procedure [30]. The precipitates were dried in a freeze drier overnight and a 36.6 mg sample was added to 500 µL of anhydrous pyridine/chloroform-d (1.6/1.0, v/v) solution. A 50 µL of a pyridine/chloroform-d (1.6/1.0) solution of chromium (III) acetylacetonate (5.6 mg/mL) was then added to the precipitates and the reaction mixture was stirred at room temperature for 10 min. A chloroform/pyridine-d mixture (1/1.6) of cyclohexanol (35 µL, 21.5 mg/mL) was then added as the internal standard. After the addition of cyclohexanol, 100 µL of 2-chloro-4,4,5,5-tetramethyl-1,2,3-dioxaphosphine was added as a phosphorylating agent and stirred for 10 min at room temperature. Upon completion, the reaction mixture was analyzed using an INOVA-500 MHz NMR instrument (Varian, USA).

## Results and discussion

### Wood chip properties

Table 1 lists the chemical compositions of spruce wood chips. It is seen that the wood chips contained 17.0, 47.2, 3.9, 2.8, and 26.5 wt% of hemicelluloses, cellulose, extractives, acid-soluble lignin, and acid-insoluble lignin, respectively. Similar compositions were reported for spruce wood chips in other reports [31, 32].

**Table 1:** Chemical compositions of spruce wood chips reported in different reports

Hemicellulose, wt%	Cellulose, wt%	Acid-soluble lignin, wt%	Acid-insoluble lignin, wt%	Extractives, wt%	Reference/origin
17.0	47.2	2.8	26.5	3.9	Current work/Canadian
18.4	45.0	–	27.6	1.0	[24]/Danish
14.8	48.0	1.0	27.2	–	[23]/Swedish

### Hydrolysis analysis

Table 2 lists the properties of hydrolysis liquor. The hydrolysis liquor produced in this work had an acidic pH of 3.2–3.6 implying that the acetyl groups attached to wood chips were partly removed and converted to acetic acid during hydrolysis process [4, 33]. It is evident that, by increasing temperature, the concentrations of monosugars and hydrolysis lignin were increased while that of polysugars was reduced. The decrease in polysugars content was ascribed to the decomposition of polysugars, which generated more monosugars in the hydrolysis liquor (Table 2) [25]. In another report, the increase in the hydrolysis temperature of poplar from 170 to 190 °C with a residence time of 60 min slightly increased the removal of monosugars from 2.4 to 4.4% [33]. The results also showed that the molecular weight of polysugars was slightly reduced by intensifying the process severity. A similar phenomenon was noted by other researchers, which indicated that this reduction could be explained by polysaccharide chains cleavage at elevated temperatures [25] or at prolonged resident times [24]. Interestingly, the furfural content has significantly increased with a temperature rise, which is consistent with the results reported previously [33]. In one study, an increase in the furfural production from the hydrolysis liquor of kraft-based dissolving pulp process was observed via increasing the temperature from 170 to 190 °C and extending residence time from 20 to 100 min [34].

**Table 2:** Properties of hydrolysis and soda liquors at different temperatures and residence times

Sample ID	Temp.,°C	Time, min	pH	Hydrolysis liquor												Soda liquor lignin		
				Mono sugar		Polysugar			Hydrolysis lignin			Furfural		Acetic acid				
				g/L	%^a^	g/L	%	Mw, g/mol	g/L	%^a^	Mw, g/mol	g/L	%^a^	g/L	%^a^	g/L	%^a^	Mw, g/mol
1	170	15	3.46	3.79	1.9	11.85	5.9	1135	11.6	5.7	21,902	0.32	0.2	1.16	0.6	0.8	0.6	2977
2	180	15	3.39	5.93	3.2	16.75	9.0	823	11.8	6.3	21,201	0.69	0.4	1.20	0.6	0.9	0.7	3239
3	190	15	3.48	12.52	6.1	7.61	3.7	526	14.3	7.0	20,166	3.86	1.9	1.32	0.6	2.7	2.1	3120
4	170	45	3.53	5.25	2.9	14.87	8.2	881	10.7	5.9	23,609	0.44	0.2	1.25	0.7	0.9	0.7	2620
5	180	45	3.37	10.70	5.8	15.89	8.7	527	11.0	6.0	22,324	1.21	0.7	1.24	0.7	1.4	1.1	2725
6	190	45	3.21	12.91	6.8	6.13	3.3	504	14.0	7.4	22,559	4.5	2.4	0.89	0.5	2.0	1.6	2786

^a^% removed from wood

The results in Table 2 also show the following: (1) by increasing the temperature at 15 min hydrolysis time, the concentration of acetic acid increased; (2) by increasing the temperature at 45 min hydrolysis time, the concentration of acetic acid decreased; and (3) by extending time of hydrolysis from 15 to 45 min, the acetic acid content of hydrolysis liquor was reduced. It is hypothesized that, at a short hydrolysis time of 15 min, more acetyl groups were cleaved by increasing temperature and thus more acetic acid was formed in the hydrolysis liquor [4]. By extending time, or basically providing more severe hydrolysis conditions, the formed acetic acid might have degraded to other products and hence the concentration of acetic acid was reduced (Table 2).

The molecular weight of the lignin in hydrolysis liquor varied between 21,000 and 24,000 g/mol. It has been found that the molecular weight of milled wood lignin, which was a representative of native lignin for spruce wood [35], was about 23,500 g/mol [36].

The results also showed that, by extending time from 15 to 45 min, there was a slight increase in the concentrations of furfural and monosugars, but a minor decrease in the concentrations of polysugars. It can be claimed that the time extension hydrolyzed (i.e., cleaved) polysugars to monosugars, but the monosugars were subsequently converted to furfural [33]. The concentration of lignin was insignificantly changed via time extension, implying that the hydrolysis conditions were not strong for major lignin removal from wood chips [5].

As discussed earlier, some lignin compounds were generated and precipitated/adsorbed to the digester surface, which were collected via soda liquor treatment. The concentration of soda liquor lignin in soda liquor is also listed in Table 2. No furfural, acetic acid, or sugars were detected in the soda liquor, indicating that these compounds did not adsorb/interact with soda liquor lignin and/or precipitate in the vessel, but were indeed remained in the hydrolysis liquor. It is evident that by increasing the temperature from 170 to 180 °C, more soda liquor lignin was removed from wood chips but the mass of removed soda liquor lignin was slightly reduced at 190 °C. The results in Table 2 show that the molecular weight of soda liquor lignin was around 3000 g/mol. The molecular weight of soda liquor lignin was lower than that of hydrolysis lignin (Table 2) and that of dioxane lignin or acetic acid-hydrolyzed lignin (6000 g/mol) reported elsewhere [37]. The molecular weight of soda liquor lignin was lower at the hydrolysis time of 45 min (compared to 15 min). Generally, by extending time, more lignin can be removed from wood chips. The reduction in molecular weight may suggest that the time extension cleaved a part of LCC intermolecular bonds and degraded some carbohydrates. Consequently, the overall molecular weight of lignin presented in soda liquor was reduced. The reduction in the amount of lignin in soda liquor at 190 °C may provide evidence for this hypothesis.

### Acidification of hydrolysis and soda liquors

Table 3 lists the properties of the hydrolysis and soda liquors after acidification (pH 1.5). Comparing the results in Tables 2 and 3 depicts that acidification has insignificantly influenced the concentrations of sugars and hydrolysis lignin, confirming that acidification was ineffective in extracting lignin or sugars from the hydrolysis liquor, and this phenomenon was due to the high solubility of hydrolysis lignin and sugars in acidic solutions. The isolation of sugars in the precipitates made from acidification of hydrolysis liquor produced at 180 °C and 45 min was due to the higher concentration of polysugars in hydrolysis liquor under these conditions (Table 2). In another work, the acidification of hydrolysis liquor of kraft-based dissolving pulp production process to pH 2 resulted in a marginal lignin and sugar separation [15], but a drop in the molecular weight of polysugars due to the hydrolysis [38]. A comparison of furfural and acetic acid concentrations before and after acidification (Tables 2, 3) shows a drop in their concentrations after acidification, which implies that these compounds were either removed from hydrolysis liquor [12] or degraded via acidification. The results in Table 3 also show that the concentration of soda liquor lignin dropped significantly after adjusting pH to 1.5.

**Table 3:** Properties of acidified hydrolysis liquor and soda liquor

Sample ID	Mono sugar^a^, g/L	Poly sugar^a^, g/L	Hydrolysis lignin^a^, g/L	Furfural^a^, g/L	Acetic acid^a^, g/L	Mw of hydrolysis lignin^a^, g/mol	Mw of poly sugar^a^, g/mol	Soda liquor lignin^b^, g/L
1	3.52	12.01	11.3	0.14	0.28	24,157	804	0.4
2	6.9	16.84	11.0	0.32	0.5	21,484	603	0.7
3	12.75	7.57	14.4	1.13	0.62	21,579	459	1.2
4	5.35	14.99	10.6	0.3	0.22	23,244	578	0.7
5	9.33	14.62	10.9	0.46	0.52	20,713	466	0.8
6	13.09	6.04	14.1	1.05	0.51	21,524	499	1.2

^a^In hydrolysis liquor

^b^In soda liquor

Generally, as more lignin and sugar were dissolved in hydrolysis and soda liquors generated at 45 min of hydrolysis treatment, these samples were selected for further analysis in this work. Table 4 shows the functional groups attached to the precipitates made from acidification of hydrolysis and soda liquors.

**Table 4:** Functional group associated with precipitates made from the acidification of hydrolysis and soda liquors generated after 45 min of the hydrolysis treatment

Temperature °C	Precipitates of hydrolysis liquor (mmol/g)			Precipitates of soda liquor (mmol/g)		
	170	180	190	170	180	190
Aliphatic OH	2.04	1.54	0.75	1.05	1.14	0.53
C_5_ substituted	0.33	0.23	0.07	0.35	0.19	0.24
Guaiacyl OH	1.42	1.61	0.94	1.22	1.14	0.86
*p*-Hydroxy phenyl	0.08	0.06	0	0.1	0.03	0.02
Carboxylic	0.14	0.14	0.06	0.71	0.67	0.53
Total	4.01	3.58	1.81	3.43	3.17	2.18

Aliphatic hydroxyl (OH), phenolate, and carboxylate groups originally exist in lignin [30, 39], and some free phenolate and aliphatic groups would be formed during hydrolysis process [40, 41]. The formation of C–C bonds would occur in the condensation reaction under acidic conditions and contributed to C–C bonds (Tables 2, 3) [42, 43]. Although Table 4 indicates a decrease in C-5 substitution, other carbon–carbon bonds, such as β-β and β-5 can be formed in the condensation reaction [44, 45]. It was reported that the precipitates of hydrolysis liquor made from steam hydrolysis of mixed hardwood contained 1.88 mmol/g of aliphatic hydroxyl groups, which agrees with that of the present work [41].

It is also evident in Table 4 that increasing the hydrolysis temperature decreased the number of functional groups in the precipitates. The decrease in the contents of the guaiacyl, phenolate, and carboxylate groups in the precipitates (Table 4) is in agreement with the molecular weight decrease at a high temperature shown in Table 2. A previous study claimed that by increasing the temperature, the degradation of carboxylate groups in spruce wood increased [46], which is consistent with the degradation of carboxylate groups in this work when the temperature increased from 170 to 190 °C. The results in Table 4 also depict that the precipitates of soda liquor had generally less aliphatic and guaiacyl hydroxyl, but more C5 substituted, carboxylate, and *p*-hydroxy phenyl groups.

### Thermal properties of precipitates from hydrolysis liquor

**Fig. 2:**
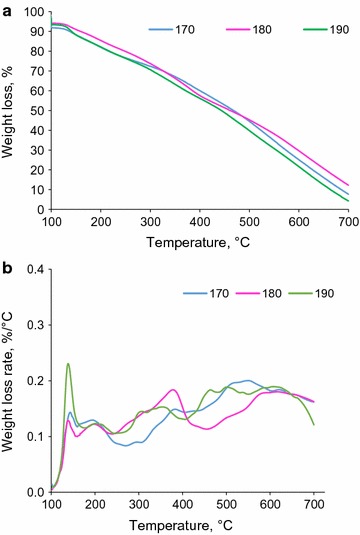
Weight loss (**a**) analysis and weight loss rate (**b**) of precipitates produced via the acidification of hydrolysis liquors generated at 170, 180, and 190 °C

The thermal analysis of precipitates was conducted to identify an end-use application for them. The weight loss of precipitates made from the acidification of hydrolysis liquor is shown as a function of temperature in Fig. 2a. It is seen that the weight loss of the precipitates was significant and only 20 wt% of precipitates were left as char at 700 °C. The weight loss rate of the samples showed a similar decomposition behavior for all samples in the temperature range of 100–700 °C (Fig. 2b). It has been reported in the literature that the pyrolysis of lignin led to 60 wt% of char [47, 48]. It is known that the pyrolysis behavior of materials depends on their compositions and structures [49]. The continuous sharp degradation of precipitates could be attributed to two reasons. (1) The precipitates contained volatile components which gradually evaporated at an increased temperature. As stated above, the concentration of furfural in hydrolysis liquor dropped via acidification and this could indirectly state that furfural precipitated via acidification (Tables 2, 3). The evaporation of furfural from precipitates would be seen as an endothermic effect in thermal analysis and contributed to the weight loss trend observed in Fig. 2. (2) Lignin was degraded in the presence of inorganic compounds at a higher temperature. Sulfuric acid has been reported to enhance the degradation of lignin in pyrolysis [50]. As precipitates were formed via sulfuric acid treatment of hydrolysis liquor, it is possible that some sulfuric acid molecules adsorbed on precipitates and hence contributed to the trend observed in Fig. 2.

**Fig. 3:**
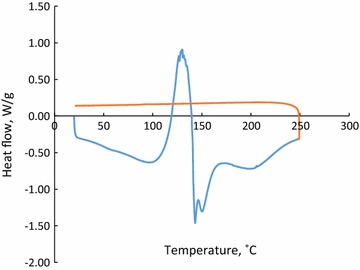
The DSC analysis of precipitates produced via acidifying of sample 1; hydrolysis at 170 °C, 15 min; *blue curve* shows heating vs. *red curve* which shows cooling

To further understand the characteristics of the precipitates, their thermal properties were analyzed by DSC. The heat flow of precipitates produced via acidifying hydrolysis liquor at 170 °C is shown in Fig. 3. The heating curve peaks are below 200 °C, the boiling temperature of acetic acid is 118 °C, and that of furfural is 161.7 °C [51]. Therefore, the sharp drop in DSC curves above 150 °C can be explained by furfural evaporation from the precipitates. A similar result was obtained for the DSC analysis of precipitates made from the acidification of hydrolysis liquor generated at 180 or 190 °C (not shown). It is inferred from Figs. 2 and 3 that precipitates made from the acidification of hydrolysis liquor contained volatile compounds that deteriorated the thermal properties of precipitates in such a way that they were not suitable as bio-fuels.

### Thermal properties of unpurified soda liquor lignin

**Fig. 4:**
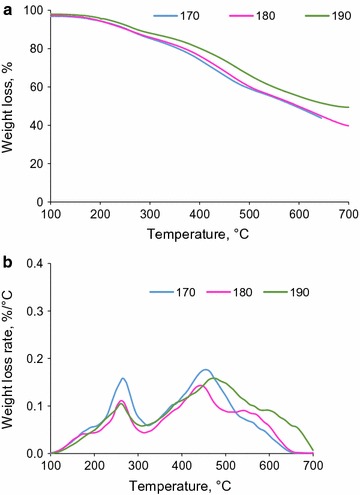
Weight loss (**a**) and weight loss rate (**b**) of unpurified soda liquor lignin of soda liquors collected at different hydrolysis temperatures (170, 180, and 190 °C) but at 45 min

Figure 4 shows the weight loss and weight loss rate of unpurified soda liquor lignin made from the acidification of soda liquor. It is seen that the first peak occurred at the same position for all the three temperatures, while the second decomposition peak occurred at a higher temperature for the sample prepared at 190 °C of hydrolysis, indicating that the precipitates would have been more thermally stable if they were produced at a higher temperature in hydrolysis process. Table 4 shows that the total amounts of functional groups are 3.43, 3.17, and 2.18 mmol/g for precipitates made from hydrolysis at 170, 180, and 190 °C, respectively. In an earlier study, the pyrolysis of wood at a temperature higher than 200 °C resulted in the degradation of carboxylate groups [46]. Similarly, the present work reveals that the high concentration of functional groups resulted in high mass loss in pyrolysis.

**Fig. 5:**
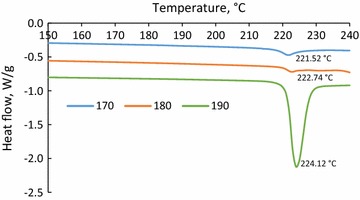
DSC analysis of the precipitates made from acidification of soda liquor generated at different hydrolysis temperatures and 45 min

Figure 5 shows the heat flow of precipitates made from the acidification of soda liquor. All precipitates showed a minimum heat flow peak (i.e., melting point) at around 220–230 °C. During the acidification of soda liquor, sulfuric acid reacted with NaOH present and forms sodium sulfite (Na_2_SO_3_) and sodium sulfate (Na_2_SO_4_). These salts would also precipitate along with lignin presented in soda liquor.

In order to verify if the endothermic peaks in Fig. 5 (i.e., melting temperature) are due to the melting of the inorganic salts in the precipitates, the heat flows of the pure salts were analyzed as functions of the temperature (Additional file 1). Among NaOH, Na_2_SO_3_, and Na_2_SO_4_, only Na_2_SO_4_ showed a peak in the heating curve at approximately 240–250 °C. The melting points of NaOH, Na_2_SO_3_, and Na_2_SO_4_ were 318, 33.4, and 884 °C, respectively [50]. An earlier study showed that the endothermic peak at about 250 °C was due to the changes in the crystalline structure of Na_2_SO_4_ [52]. This confirmed that the peak for Na_2_SO_4_ was a consequence of the changes in the structure of Na_2_SO_4_ present in soda liquor lignin. Therefore, the results confirmed that the heat flow peaks in Fig. 5 were not generated by the organics of precipitates, but they were created by inorganic compounds (i.e., sodium sulfate) present in the precipitates.

### Thermal properties of purified soda liquor lignin

**Fig. 6:**
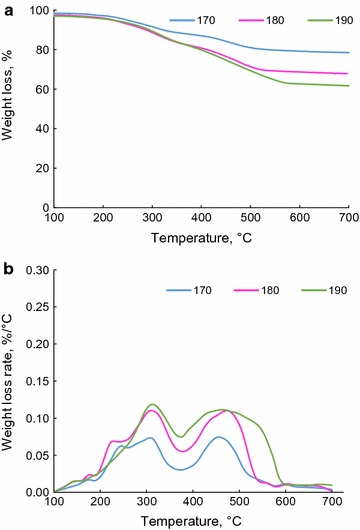
Weight loss (**a**) and weight loss rate (**b**) of purified soda liquor lignin of soda liquor collected at different hydrolysis temperatures (170, 180, and 190 °C) but at 45 min

To remove inorganic salts from soda liquor lignin, the precipitates made from acidification of soda liquor were dialyzed in water with membrane. Figure 6a shows the weight loss of the precipitates after dialysis. It is seen that the pyrolysis of the precipitates resulted in 60 wt% char, and the precipitates made from hydrolysis at 190 °C generated less char than those made at a lower temperature. It can be seen in Fig. 6b that the samples produced via hydrolysis at 170 and 180 °C started to decompose at the lower temperature of 200 °C; however, the sample prepared via hydrolysis at 190 °C decomposed when the temperature was higher than 300 °C. The comparison between the results in Figs. 4 and 6 also depicts that after the pyrolysis, the final char content was higher in the purified precipitates than in the unpurified precipitates. This suggests that membrane dialysis removed some low molecular weight organic compounds in addition to inorganics. Therefore, after pyrolysis of the ash-free lignin, more char was formed.

It is worth noting that the mass loss in Fig. 6 (20–40 wt%) is less than the typical mass loss for the pyrolysis of lignin (40–50%). As discussed earlier, to remove inorganic salts in the dialysis process, some of the small molecular weight compounds might have passed through the membrane. Accordingly, the purified precipitates (i.e., purified soda liquor lignin) had a higher molecular weight, and thus showed higher thermal stability (i.e., less mass loss).

The heat flow of purified precipitates was also analyzed as a function of temperature (Additional file 1). A peak in the heat flow was observed at 180–190 °C, which suggested that the structure of precipitates changed its state from a glassy state into a rubbery state. The Tg of lignin depends on the processes by which lignin is produced and on the wood source [53]. It has been reported that Tg of lignin made from acid hydrolysis of softwood was 95 °C, while that of lignin made from steam explosion of softwood was 139 °C [54]. In another report, the Tg of lignin made from hot water hydrolysis of hardwood was 170–180 °C [55]. In this work, increasing the temperature of hydrolysis treatment from 170 to 190 °C increased the Tg from 184 to 192 °C. This is consistent with an earlier study in which a Tg increase by 8 °C was reported for increasing the temperature of hot water treatment of hardwood by 20 °C [55]. Also, heat flow analysis of purified precipitates did not experience any melting point between 200 and 220 °C, which confirms that the melting points in Fig. 4 were indeed due to the presence of inorganic salts in the impure precipitates.

### Process development and product applications

Following the steps shown in Fig. 1, mass balance was developed to identify how much hot water hydrolysis would remove different components from wood chips and the results are listed in Table 5. It is observable that approximately 25% of wood chips were isolated via hydrolysis. In the literature, the temperature of 180 °C and time of 2 h reported to dissolve more components than a higher temperature and longer time in the hydrolysis process, which support the results presented in Table 5 [56]. The summation of organic compounds in the hydrolysis liquor after acidification, the precipitates of acidification and soda liquor lignin were approximately 95–98%, which shows the high validity of the analysis. The results also depict that acidification of hydrolysis liquor reduced the acetic acid and furfural contents of hydrolysis liquor. Saeed et al. stated that acetic acid and furfural shared 1.31–1.52 and 0.18–0.31% of the total solid content of industrially produced hardwood hydrolysis liquor [4]. In the present work, acetic acid and furfural contents of softwood hydrolysis liquor ranged from 0.5 to 0.7 wt% and from 0.2 to 2.4 wt% based on the total solid content of hydrolysis liquor, respectively. The discrepancy could be attributed to the differences in the raw materials used in these two studies. Therefore, acidification of hydrolysis liquor (rather than energy intensive evaporation) can be proposed as a method to isolate volatile compounds. However, most polysugars, monosugars, and hydrolysis lignin remained in the solution. Interestingly, the amount of lignin adsorbed on the digester (and thus removed via soda treatment) was higher than that removed via acidifying hydrolysis liquor.

**Table 5:** Mass of components in treated wood chips, precipitates of acidification of hydrolysis liquor, acidified hydrolysis liquor, acidified soda liquor, and soda liquor lignin (based on 100 g oven dried wood)

Sample ID	Acidified hydrolysis liquor, %^a^					Precipitates of acidified hydrolysis liquor, %^a^					Acidified soda liquor, %^a^		Wood residue, %	Total, %
	Mono sugar	Poly sugar	Hydrolysis lignin	Furfural	Acetic acid	Mono sugar	Poly sugar	Hydrolysis lignin	Furfural	Acetic acid	Lignin in solution	Lignin precipitated		
4	2.9	8.6	5.8	0.1	0.1	NA	NA	0.1	0.1	0.6	0.5	0.2	76.7	95.7
5	5.1	8.0	5.9	0.3	0.3	0.7	0.7	0.1	0.5	0.4	0.6	0.5	75.1	98.2
6	6.9	3.3	7.5	0.6	0.2	NA	NA	NA	1.9	0.3	1.0	0.6	74.3	96.6

^a^% removed from wood

**Fig. 7:**
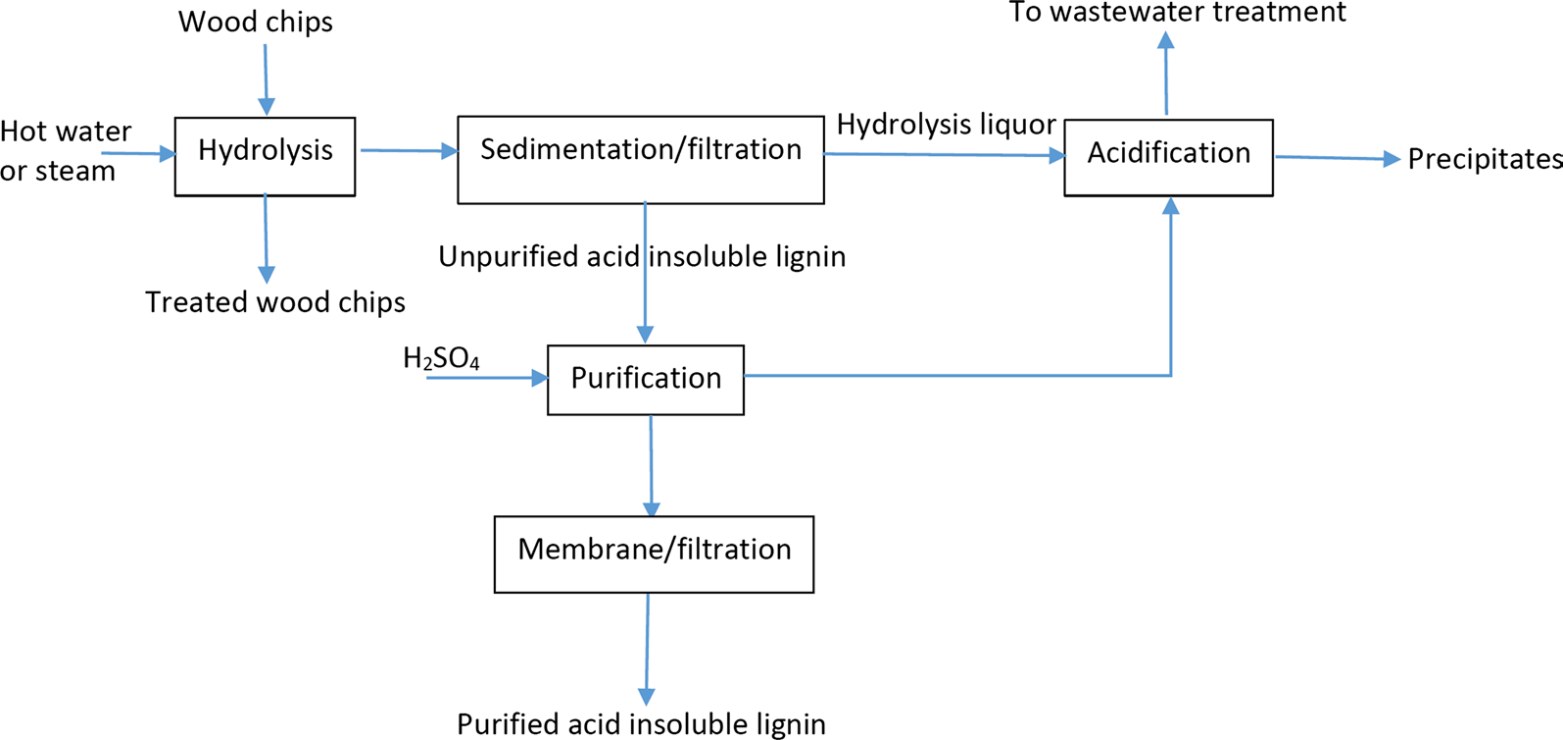
Process for producing hydrolysis lignin and precipitates

Figure 7 presents a process for extracting organic materials from hydrolysis liquor. The results in this work show that the hydrolysis of wood chips leads to the extraction of mono and polysugars, furfural, acetic acid as well as lignin from wood chips. As stated earlier, some less soluble lignin compounds were formed and adsorbed on the digester’s interior surface. In a large-scale operation, a part of these compounds would be adsorbed on the digester. After saturation of the digester’s surface, these compounds would not be able to adsorb on the digester due to the shear stress and pressure applied in the hydrolysis process from feeding/discharging of the digester as well as circulating the hydrolysis liquor in the hydrolysis process as conducted in industry [5]. Therefore, these compounds will be coagulated and form lignin compound flocs. A sedimentation/filtration process could facilitate the precipitation of these lignin flocs from the system. To have purified lignin flocs, the unpurified hydrolysis lignin separated via sedimentation/filtration can be further washed with acids, e.g., sulfuric acid, and then purified with a membrane. The organics can be isolated from the hydrolysis liquor by heating and acidifying to pH 1.5. Heating is claimed to be a key element in the formation and coagulation of kraft lignin in the LignoForce technology [16]. The results in Tables 2, 3, and 5 show that the acidification of hydrolysis liquor can be used as means to extract volatile compounds (e.g., furfural and acetic acid) from hydrolysis liquor with precipitates of 2% based on wood mass (Table 5).

Afterward, the acidified hydrolysis liquor can be sent to wastewater treatment. It can then be neutralized and further treated via biological treatment processes [57]. In the developed process shown in Fig. 7, the spent acid that is generated in the purification process of lignin can be used as a source of acid for acidification of hydrolysis liquor. This process is similar to LignoForce in that it uses sedimentation/filtration tank to isolate lignin compounds from hydrolysis liquor and it uses acid washing to further purify the lignin compounds.

The thermal analyses confirm that soda liquor lignin had thermally stable characteristics. Therefore, this product can be used in composite production (e.g.. lignin–epoxy resin composites) in which the thermal stability of composite is an advantage [58]. However, more in-depth analysis is required to validate it. In addition, the acidification of hydrolysis liquor made the precipitates that were mainly furfural and acetic acid. These chemicals can be used as platform chemicals for the production of other value-added chemicals, such as plastics, pharmaceutical, and agrochemical industries [59].

As seen in Table 5, it would be possible to produce 1, 5, and 19 tons of furfural from 1000 tons of wood chips if the procedures for the production of samples 4, 5, and 6 were followed, respectively. Acetic acid would be produced at the rates of 6, 4, and 3 tons if the procedures for the samples 4, 5, and 6 were followed, respectively. It should be stated that this process may not be economical if considered independent as the extraction yield is rather low (Table 5). However, hydrolysis is commercially used as a pretreatment step in the production of dissolving pulp following kraft process and is used for producing wood pellets [4–6]. The proposed process may be economical in these processes, as it may generate additional revenues from a waste resource. However, detailed economic analysis should be conducted to understand the financial feasibility of the proposed process.

## Conclusions

Processing temperature and residence time showed major effects on the furfural production, but minor effects on sugars and lignin isolations from treated biomass. Increasing the severity of hydrolysis process led to increases in the concentrations of hydrolysis lignin from 11.6 to 14.3 g/L, monosugars from 3 to 12 g/L, and furfural from 0.3 to 4 g/L. However, the concentration of polysugar was the maximum at 180 °C, and more sever conditions reduced its concentration in hydrolysis liquor. The molecular weight of hydrolysis lignin was approximately 22 kg/mol and was insignificantly changed under different hydrolysis conditions. The acidification of hydrolysis liquor led to the precipitates that contained mainly furfural and acetic acid rather than hydrolysis lignin and sugars. The concentrations of furfural and acetic acid in hydrolysis liquor were significantly decreased (approximately 31–76%), but those of lignin and sugars reduced by less than 13%. The acidification of soda liquor led to precipitation of soda liquor lignin (1% removed from the wood chips) and inorganic impurities. The purified soda liquor lignin showed Tg around 180–190 °C and thermal resistance. Based on these results, a process for producing purified lignin and the precipitates of volatile compounds from hydrolysis liquor was developed.

## Additional file

**Additional file 1: Figure S1.** DSC analysis of a) NaOH, b) Na_2_SO_3_ and c) Na_2_SO_4_ (heating curve is blue). **Figure S2.** Heat flow of purified precipitates made from soda liquor.

## Abbreviations

DSC: differential scanning calorimetry
LCC: lignin–carbohydrate complexes
L/S: liquid to solid
NMR: nuclear magnetic resonance
TGA: thermogravimetric analysis
TPS: 3-(trimethylsilyl) propionic-2,2,3,3-d_4_ acid sodium salt
