# Supplementary material for: A process for producing lignin and volatile compounds from hydrolysis liquor
Source: Biotechnol Biofuels. 2017 Feb 23;10:47. doi: 10.1186/s13068-017-0729-9 (PMC5322682; doi:10.1186/s13068-017-0729-9)
Supplement: Supplementary file 1 — Additional file 1: Figure S1. DSC analysis of a) NaOH, b) Na2SO3 and c) Na2SO4 (heating curve is blue). Figure S2. Heat flow of purified precipitates made from soda liquor. [file 13068_2017_729_MOESM1_ESM.docx]

Supplementary figures

Figure S1: DSC analysis of a) NaOH, b) Na_2_SO_3_ and c) Na_2_SO_4_ (heating curve is blue)


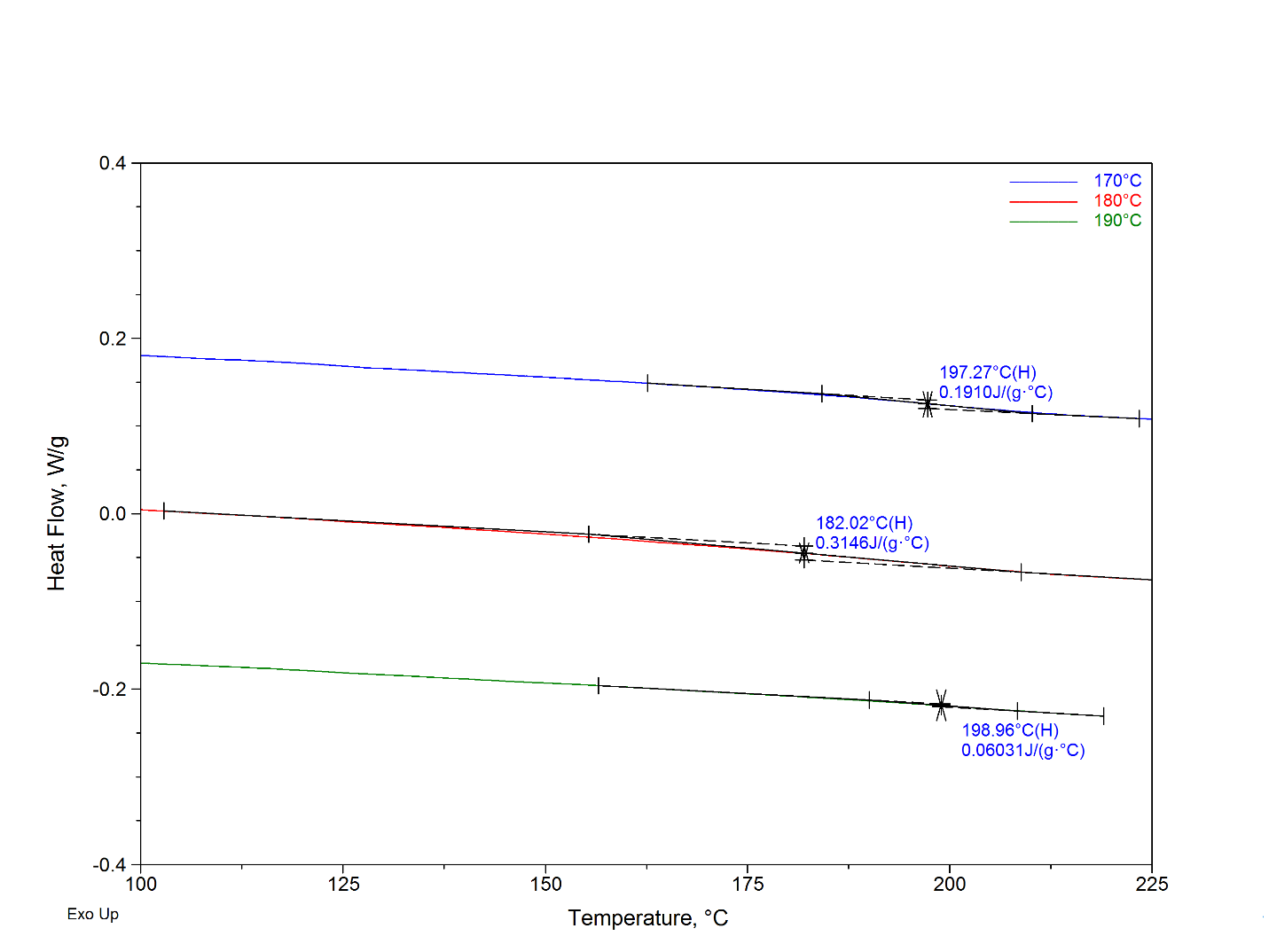


Figure S2: Heat flow of purified precipitates made from soda liquor
